# The Out-of-Plane Compression Response of Woven Thermoplastic Composites: Effects of Strain Rates and Temperature

**DOI:** 10.3390/polym13020264

**Published:** 2021-01-14

**Authors:** Shiyu Wang, Lihua Wen, Jinyou Xiao, Ming Lei, Xiao Hou, Jun Liang

**Affiliations:** 1 School of Astronautics, Northwestern Polytechnical University, Xi’an 710072, China; wangsy@nwpu.edu.cn (S.W.); lhwen@nwpu.edu.cn (L.W.); Leiming@nwpu.edu.cn (M.L.); houxiaoht@163.com (X.H.); 2 Institute of Advanced Structure Technology, Beijing Institute of Technology, Beijing 100081, China

**Keywords:** thermoplastic composites, mechanical properties, strain-rate effects, failure mechanism

## Abstract

The dynamic mechanical response of high-performance thermoplastic composites over a wide range of strain rates is a challenging research topic for extreme environmental survivability in the field of aerospace engineering. This paper investigates the evolution of the dynamic properties of woven thermoplastic composites with strain rate and damage process at elevated temperatures. Out-of-plane dynamic-compression tests of glass-fiber (GF)- and carbon-fiber (CF)-reinforced polyphenylene sulfide (PPS) composites were performed using a split Hopkinson pressure bar (SHPB). Results showed that thermoplastic composites possess strain-rate strengthening effects and high-temperature weakening dependence. GF/PPS and CF/PPS composites had the same strain-rate sensitivity (SRS) below the threshold strain rate. The softening of the matrix at elevated temperatures decreased the modulus but had little effect on strength. Some empirical formulations, including strain-rate and temperature effects, are proposed for more accurately predicting the out-of-plane dynamic-compression behavior of thermoplastic composites. Lastly, the final failure of the specimens was examined by scanning electron microscopy (SEM) to explore potential failure mechanisms, such as fiber-bundle shear fracture at high strain rates and stretch break at elevated temperatures.

## 1. Introduction

There is strong interest in accurately describing the mechanical response of high-performance thermoplastic composite structure at high strain rates in aerospace-engineering applications [1,2,3,4,5]. To adequately understand the evolution of dynamic properties with strain-rate and damage processes, experimental data on the mechanical properties of thermoplastic composites under high-strain-rate compressive loads in service environments are required. Composite structures are greatly sensitive to environmental temperature, especially for dynamic problems [6,7,8,9,10]. Thus, it is essential to investigate the mechanical properties and failure mechanisms of thermoplastic composites under dynamic loads at elevated temperatures.

Considerable attention was paid upon characterizing the dynamic behavior of composite in the strain rate range of 100–10,000/s using a split Hopkinson pressure bar (SHPB) [11,12,13,14,15]. Tarfaoui et al. [11] carried out inplane and out-of-plane high-strain-rate compressive behavior of angle-ply plain weave composite laminates, and found that the dynamic behavior of the composite was sensitive to fiber orientation and loading direction. Similar work was performed by Kara et al. [12], Hosur et al. [13], and Song et al. [14]. In these studies, the peak stress and modulus of the composite in both directions increased with the increase in strain rate, while strain-rate sensitivity in the inplane direction was more noticeable than that in the out-of-plane direction. In addition, Woldenbet and Vinson [15] experimentally studied the effect of specimen geometry on the high-strain-rate compressive behavior of graphite/epoxy laminates, and indicated that the difference in the experiment results of cube and cylinder specimens was not apparent. The above-mentioned dynamic compressive responses of fiber-reinforced composites were mainly focused on thermoset composites. There are few studies on thermoplastic composites [6,10,16,17,18].

Montes et al. [16], and Brown et al. [17] found that the high-strain-rate inplane compressive strength of thermoplastic composites significantly increased in comparison with quasistatic compressive strength, while an opposite conclusion was reported by Qian et al. [6] for the out-of-plane compressive strength of aramid-fabric-reinforced polyamide composite. Massaq et al. [18] conducted quasistatic and dynamic-compression testing on a PA6/glass composite in three directions and found that strain-rate sensibility depended on compressive direction. Recently, Wang et al. [10] further studied the mechanical properties and failure modes of a carbon-fiber (CF)/polyphenylene sulfide (PPS) composite in the inplane and out-of-plane directions with different strain rates in the range of 350–1550/s, while the effect of temperature on the failure mechanisms of the CF/PPS composite was not studied in detail.

Although thermoplastic composites were extensively characterized under quasistatic tensile, compressive, shear, and fatigue loading conditions [19,20,21,22,23,24,25,26,27], few studies concerned the dynamic mechanical behavior of thermoplastic composites at elevated temperatures. Thermoplastic composites are significantly sensitive to temperature. The matrix softening results in a decrease in the modulus and strength of composites, especially above the glass-transition temperature [28,29,30]. Schoßig et al. found that the dynamic tensile strength and modulus of thermoplastic composites decreased with the increase in temperature [31]. Barba et al. [32] also expounded that thermoplastic composites have strain-rate enhancement and high-temperature weakening dependence via the dynamic tensile testing of a CF/PEEK composite. In general, the coupling effect of strain rate and temperature on the mechanical properties of composites is more complicated. Thus, there is a lack of systematic research on the dynamic-compression behavior of thermoplastic composites at elevated temperatures.

In this paper, the evolution of dynamic properties of woven thermoplastic composites with strain rate and damage process at elevated temperatures is investigated. The effects of strain rate and temperature on the out-of-plane dynamic mechanical properties and failure mechanisms of thermoplastic composites are studied. High-strain-rate out-of-plane compression tests for two kinds of woven composites (CF/PPS and glass fiber (GF)/PPS) were conducted over a wide range of temperature (23–150 °C) and strain rate (650–3500/s). The damage and fracture morphologies of specimens after failure of these two kinds of composites were analyzed to reveal their failure mechanisms. Some empirical formulations, including strain rate and temperature effects, are proposed for predicting the out-of-plane dynamic compression responses of these two kinds of composites. This study is helpful in comprehensively understanding the dynamic mechanical properties of thermoplastic composites and guiding their engineering application.

## 2. Materials and Experiments

### 2.1. Materials

**Figure 1:**
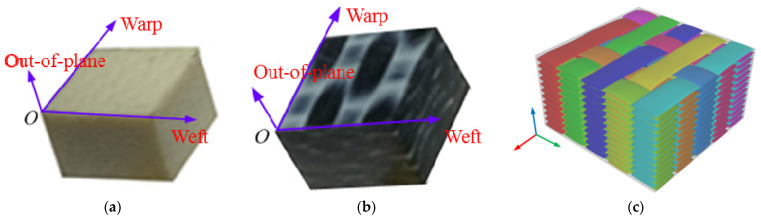
Thermoplastic composite specimens and weave construction. (**a**) Glass-fiber (GF)/ polyphenylene sulfide (PPS) specimen; (**b**) carbon-fiber (CF)/PPS specimen; (**c**) weave construction.

The thermoplastic woven composites used in this study were provided by TenCate Advanced Composites Company. The matrix material was semicrystalline high-performance PPS (Fortron 0214 PPS), and the reinforcing fibers were 5 satin glass-fiber fabric (7781) and carbon-fiber fabric (T300). Fiber volume fractions in the composite were 52.5% and 50%, respectively. The composite laminates were hot-pressed according to the lay-up sequence of ((0.90))_12_. Laminates were then cut into 6 (length) × 6 mm (width) cube specimens through water cutting, as shown in Figure 1. The thickness of the GF/PPS and CF/PPS specimen was 3.80 and 3.78 mm, respectively. The porosity of both specimens was less than 1%. To avoid the size effects in the impact tests, the specimens in all tests had the same size. The faces of the specimens were polished with 1000 grit sandpaper to ensure parallel loading edges.

### 2.2. Experiments

**Figure 2:**
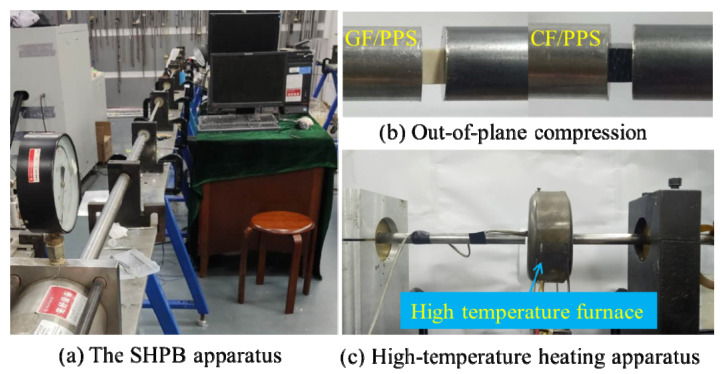
Experiment equipment and specimen assemblage scheme. (**a**) The SHPB apparatus; (**b**) Out-of-plane compression; (**c**) High-temperature heating apparatus.

Dynamic-compression tests were performed using the split Hopkinson bar (SHPB) system as shown in Figure 2, where bar diameter was 12.7 mm. A high-temperature furnace equipped with a thermocouple was used to heat the specimen during tests. Dynamic-compression tests were conducted in four temperature points (23, 90, 120, and 150 °C). The SHPB system mainly included a gas chamber, a titanium pulse guide bar, an aluminum alloy incident bar, an aluminum transmitted bar, and a striker bar. The lengths of the aluminum alloy striker, incident, and transmission bars were 200, 1200, and 1200 mm, respectively. The specimen was sandwiched between incident and transmission bar. When the striker bar was released by nitrogen gas at different pressure levels, the striker bar accelerated at different rates and impinged on the incident bar to generate different strains and strain rates in the specimen. Detailed theory and the technique involved in SHPB are well-described in the literature [33,34]. The incident, reflected, and transmitted waves were recorded by oscilloscope in terms of voltages $V_{i}$, $V_{r}$, and $V_{t}$ by the working principle of the strain gauge (with resistance of 999.0 ± 1.0 ohm and gage factor of 1.90 ± 1%). These values were converted into strain signals, including transmitted strain signal $\varepsilon_{t}$ and reflected strain signal $\varepsilon_{r}$ by using the following equations [35]:

(1) $$\varepsilon_{r}=\frac{2V_{r}}{GUA}$$

(2) $$\varepsilon_{t}=\frac{2V_{t}}{GUA}$$

where *G* is gage factor, *U* is input voltage, and *A* is amplification factor.

On the basis of one-dimensional stress wave theory, stress (σ), strain (ε), and strain rate ($\dot{\varepsilon }$) are described by the following equations [36]:

(3) $$\sigma \left(t\right)=\frac{A_{b}}{2A_{S}}E_{b}\left(\varepsilon_{i}+\varepsilon_{r}+\varepsilon_{t}\right)$$

(4) $$\dot{\varepsilon }\left(t\right)=\frac{C_{b}}{L_{S}}\left(\varepsilon_{i}-\varepsilon_{r}-\varepsilon_{t}\right)$$

(5) $$\dot{\varepsilon }\left(t\right)=\frac{C_{b}}{L_{S}}\int_{0}^{t}\left(\varepsilon_{i}-\varepsilon_{r}-\varepsilon_{t}\right)dt$$

where $A_{b}$, $A_{S}$, and $L_{S}$ are the cross-sectional area of the pressure bar, the original cross-sectional area, and the initial length of the specimen, respectively. $E_{b}$ and $C_{b}$ are the modulus and the elastic wave speed of the pressure bars, $C_{b}=\sqrt{E_{b}/\rho_{b}}$.

There were at least three specimens for each loading case with the same impact pressure and environmental conditions. The microscopic characteristics of the thermoplastic composites after dynamic tests were examined to study dynamic compressive damage and failure mechanisms.

## 3. Results and Discussion

### 3.1. Dynamic Compressive Behavior

**Figure 3:**
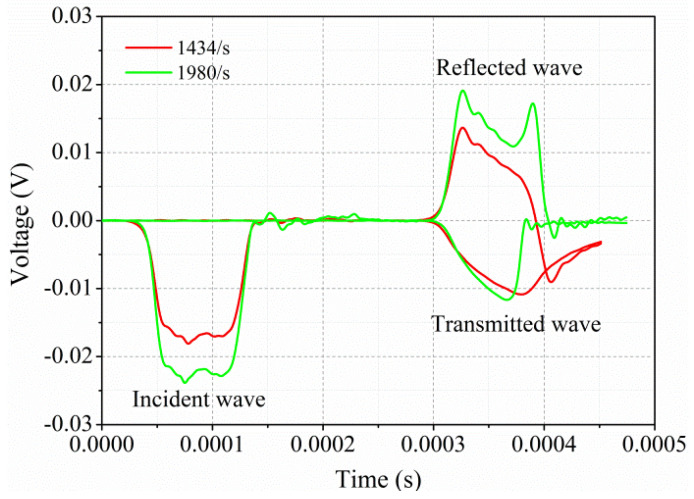
Original signals of incident, reflected, and transmitted waves recorded by strain gauges.

Figure 3 illustrates the original signals of incident, reflected, and transmitted waves in an SHPB test on GF/PPS specimens at strain rates of 1434 and 1980/s. The size of incident and transmitted pulse varied with the increase of impact pressure. The second peak of the reflected pulse and shortening the shape of the transmitted wave resulted in the presence of macroscopic damage [11,37,38]. At a strain rate of 1434/s, there was no second peak in the reflected curve, while it existed for 1980/s. The triangular shaped region of the reflected pulse indicated that the strain rate was not exactly constant for high speed tests. The indicated value of strain rate was an average value. Furthermore, the strain-gage signal from the transmission bar was converted into stress, and the reflected strain pulse from the incident bar was converted into strain and strain rate using Equations (1)–(5), respectively. Strain versus time and stress versus time responses were obtained and superimposed to obtain a dynamic stress–strain response. The effects of strain rate and temperature on dynamic-compression properties in terms of peak stress, strain at peak stress, and the slope of the stress–strain response are discussed and analyzed in the following sections.

#### 3.1.1. Effect of Strain Rate on Out-of-Plane Dynamic Compressive Response

**Figure 4:**
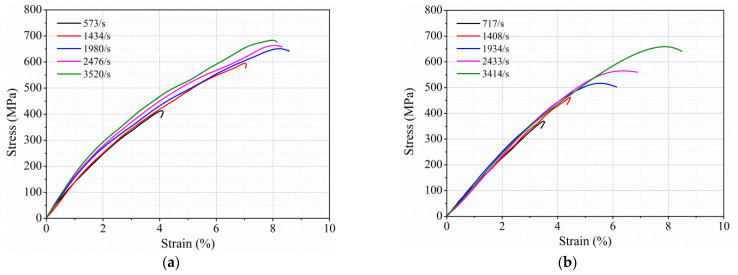
Stress–strain curves of composites under out-of-plane loading at room temperature (RT). (**a**) GF/PPS and (**b**) CF/PPS specimens.

The effect of strain rate on the out-of-plane dynamic-compression response of thermoplastic composites was investigated, shown in Figure 4, which provides the stress–strain curves of GF/PPS and CF/PPS specimens under five strain rates at room temperature (RT). As shown in Figure 4a, the stress–strain curves have different characteristics with the increase in strain rate. At lower strain rates (573 and 1434/s), GF/PPS specimens were undamaged with minimal plastic deformation, which was indicated by a hysteresis loop at the end of the stress–strain curve. With the increase in strain rate (1980/s), a nearly vertical drop of the stress–strain curve at failure reflected the macroscopic damage of the GF/PPS specimen. Therefore, a threshold strain rate of GF/PPS specimens was between 1434 and 1980/s, beyond which catastrophic failure occurred. The failure strain decreased with the increase in strain rate due to the brittle failure of the glass-fiber bundle within a short impact time. Similar behavior was also reported by Kara for the high-strain-rate compression response of the E-glass/polyester composite [12].

Stress–strain curves in Figure 4b also illustrate that strain rate has an important influence on the out-of-plane dynamic compressive response of CF/PPS composites. A threshold strain rate of CF/PPS specimens was between 1408 and 1934/s. Below the threshold strain rate, the stress–strain curves were approximately linear, corresponding to elastic behavior. Once the threshold strain rate had been exceeded, the macroscopic failure of the CF/PPS specimen manifested as a nonlinear curve and abruptly declined at the end of the curve. The phenomenon can be attributed to thermal softening due to inelastic heat dissipation and damage [39]. Unlike GF/PPS composites, within the strain-rate range of 717–3414/s, the peak stress and corresponding failure strain of the CF/PPS composites increased significantly with the increase in strain rate.

**Figure 5:**
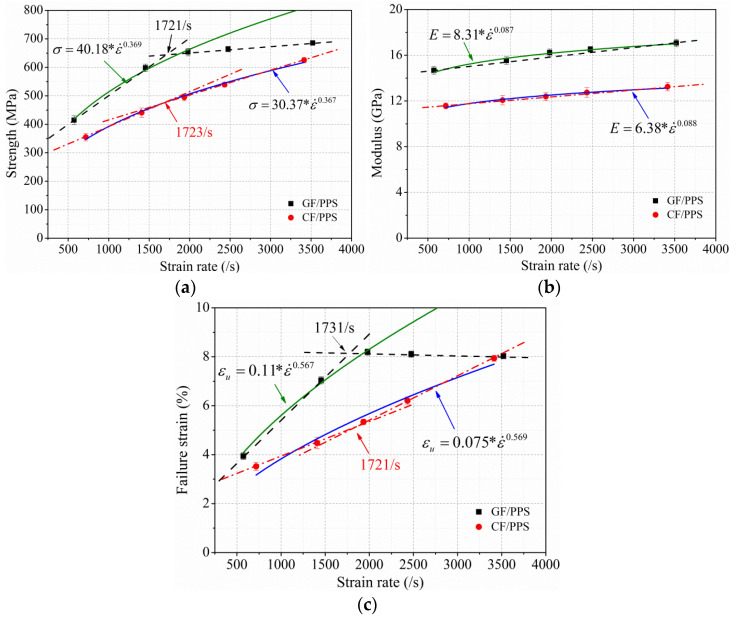
Dynamic properties of thermoplastic composites in out-of-plane direction at various rates. (**a**) Strength; (**b**) modulus; (**c**) failure strain.

In order to investigate the effect of strain rate on the out-of-plane dynamic compressive properties of thermoplastic composites, the average out-of-plane dynamic compressive strength, modulus, and failure strain of GF/PPS and CF/PPS composites at RT in Table 1 are discussed and plotted in Figure 5. The strength, modulus, and failure strain of these two thermoplastic composites increased linearly with the increase in strain rate until a strain rate threshold had been reached. There was an inflection point in the properties of the thermoplastic composites with the change in strain rate that corresponded to the threshold strain rate, especially for GF/PPS composites. From the tangent of the experiment data in Figure 5a–c, the threshold strain rates of the two thermoplastic composites were both at around 1730/s. After exceeding the threshold strain rates, the strength of the GF/PPS composites did not significantly increase with the increase in strain rate, while failure strain gradually decreased. This may be attributed to the brittle failure of the specimen in a very short impact time under higher strain rate. In addition, the strength, modulus, and failure strain of CF/PPS composites increased approximately linearly when strain rate increased from 717 to 3414/s.

In order to assess the effect of strain rate on the out-of-plane dynamic compressive properties of thermoplastic composites, a well-known Backofen formula [40] was applied to analyze the experiment data:

(6) $$\sigma =K_{\sigma }*{\dot{\varepsilon }}^{m},$$

where σ denotes stress, $\dot{\varepsilon }$ is strain rate, $K_{\sigma }$ is an intrinsic parameter of the composite, and m is the strain-rate-sensitivity (SRS) index expressed as

(7) $$m=\frac{\mathrm{In}\left(\sigma /\sigma^{0}\right)}{\mathrm{In}\left(\dot{\varepsilon }/{\dot{\varepsilon }}^{0}\right)}$$

The strength, modulus, and failure strain of the GF/PPS and CF/PPS composites are plotted in Figure 5 as a function of strain rate. Material parameter $K_{i}$ and SRS index m were obtained by power law function fitted to the experiment values as listed in Table 2. As shown in Figure 5, the fitting curve of CF/PPS specimen agreed with the compressive properties, while the fitting curve of GF/PPS specimen could only accurately predict the strength and failure strain before the threshold strain rate. All fitting curves of the compressive properties showed an obvious increasing tendency with strain rate, while the increasing amplitude was different. Comparing parameters $K_{i}$ and SRS index m in Table 2 shows that the $K_{i}$ of GF/PPS composites were all higher than those of CF/PPS composites, which reflects the better impact resistance of GF/PPS composites. Similar results were reported by Naik [41]. SRS index m reflects the sensitivity of material properties to strain rate, and the same m value of GF/PPS and CF/PPS composites indicates that the SRS index of out-of-plane dynamic compression properties may be dominated by PPS matrix.

#### 3.1.2. Effect of Temperature on Out-of-Plane Dynamic Compressive Response

**Figure 6:**
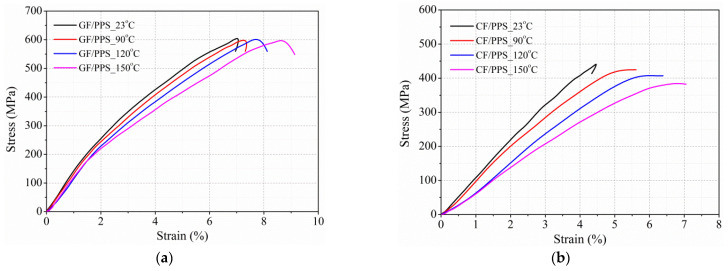
Stress–strain curves of composites under out-of-plane loading at different temperatures. (**a**) GF/PPS and (**b**) CF/PPS specimens.

Figure 6 illustrates the out-of-plane dynamic stress–strain behavior of these two kinds of thermoplastic composites at a strain rate of 1434/s and four temperature levels (RT, and 90, 120, and 150 °C). The stress–strain curves of thermoplastic composites at different temperatures had similar trends before peak stress, and the curves then showed different trends corresponding to the different failure modes of the composites. For GF/PPS specimens, curves ending at RT and 90 °C had hysteresis curves, reflecting that the composites had no macroscopic failure. Moreover, sharp-drop curves at 120 and 150 °C indicated that the strain-rate threshold decreased with increasing temperature. For the CF/PPS specimens, macroscopic failure occurred at 90 °C, indicating that the CF/PPS composites were more sensitive to temperature.

**Figure 7:**
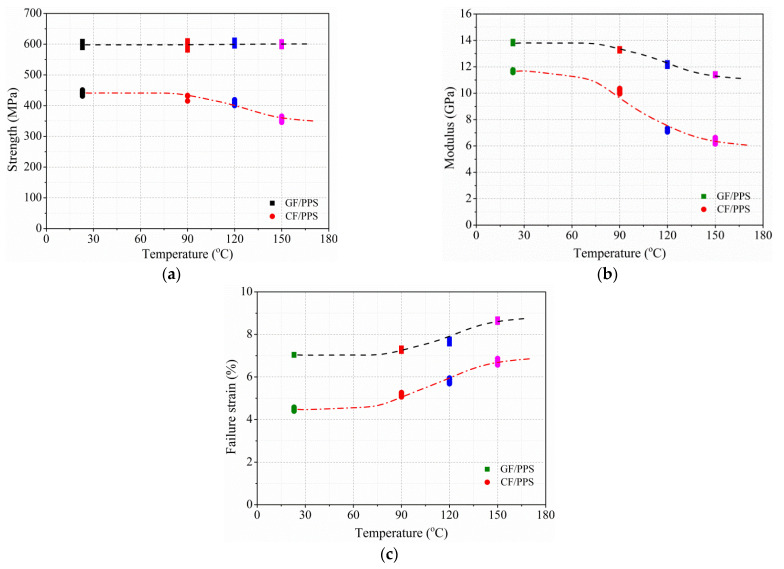
Dynamic properties of two thermoplastic composites in out-of-plane direction at different temperature levels (at 1450/s). (**a**) Strength; (**b**) modulus; (**c**) failure strain.

The above discussion shows that the out-of-plane dynamic-compression behavior of thermoplastic composites is sensitive to temperature. The average out-of-plane dynamic compressive strength, modulus, and failure strain of the GF/PPS and CF/PPS composites are summarized in Table 3 and plotted in Figure 7. Figure 7a shows that the out-of-plane dynamic compressive strength of GF/PPS composites did not change with increasing temperature. The phenomenon can be attributed to out-of-plane compressive strength mainly dominated by fiber breakage, which is not sensitive to temperature (RT, 150 °C). However, the strength of CF/PPS composites decreased slightly. This was mainly due to the softening of the matrix at elevated temperatures, which significantly reduced the load transfer capacity between layers and the stiffness of the CF/PPS composite, as shown in Figure 7b, resulting in a reduction in strength. Similarly, the softening of the matrix led to the enhancement of toughness and the increase in plastic deformation under dynamic out-of-plane compressive load, as displayed in Figure 7c.

A typical thermomechanical model from our former work [23] was applied in this study to predict the mechanical properties of thermoplastic composites at different temperatures.

(8) $$P\left(T\right)=P_{T_{0}}\frac{E^{\prime }\left(T\right)-E^{\prime }\left(T_{r}\right)}{{E^{\prime }}_{T_{0}}-{E^{\prime }}_{T_{r}}}+P_{T_{r}}\frac{{E^{\prime }}_{T_{0}}-E^{\prime }\left(T\right)}{{E^{\prime }}_{T_{0}}-{E^{\prime }}_{T_{r}}}$$

where P(T) is the mechanical property of the composite at temperature T, $P_{T_{0}}$ is the mechanical property at room temperature, and $P_{T_{r}}$ denotes the mechanical property at another reference temperature $T_{r}$, $E^{\prime }\left(T\right)$ is storage stiffness at temperature T, and ${E^{\prime }}_{T_{0}}$ and ${E^{\prime }}_{T_{c}}$ are storage stiffness at reference temperatures $T_{0}$ and $T_{r}$.

(9) $$MAPE=\sum_{t=1}^{n}\left|\frac{observed_{t}-predicted_{t}}{observed_{t}}\right|\times \frac{100}{n}$$

**Figure 8:**
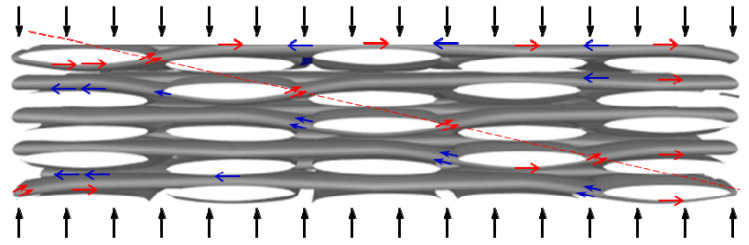
Illustration of out-of-plane compression failure mechanism of woven thermoplastic composites.

In this paper, 120 °C was selected as the reference temperature, and forecast curves are shown in Figure 7. Figure 8 shows that the forecast curves were able to replicate a variation pattern of the experiment results. Mean absolute percentage errors (*MAPE*) as Equation (9) were introduced to evaluate the accuracy of forecasting models as listed in Table 4. The value in Table 4 reflects that the maximal *MAPE* did not exceed 2.5%, which further illustrates the accuracy of the forecast model.

### 3.2. Fracture Morphologies and Failure Mechanisms of Thermoplastic Composite under Out-of-Plane Dynamic Compression

Figure 8 schematically illustrates the potential failure mechanisms of woven thermoplastic composites under out-of-plane compressive loading. The impact wave propagated along each layer, and the fiber bundles at the interweave area were subjected to high shear stress because of the fiber-weaving structure. Simultaneously, the warp-fiber bundles underwent extension due to the Poisson effect. Hence, fiber bundles at the interweave area bore the coupling effect of shear and tensile stresses. Once the fiber bundles had undergone tensile fracture or shear failure, the matrix in and around the fiber bundles also developed cracks. Furthermore, stress redistribution among intact fiber bundles resulted in higher stress, which caused more fiber fractures and interfacial crack propagation. A series of failures took place in the approximate structure of the specimen to form a failure plane. As the impact wave increased, more damage mechanisms were involved, from microcracks to macrocracks with multiple paths to the final fracture.

#### 3.2.1. Effect of Strain Rates on Failure Mechanisms

**Figure 9:**
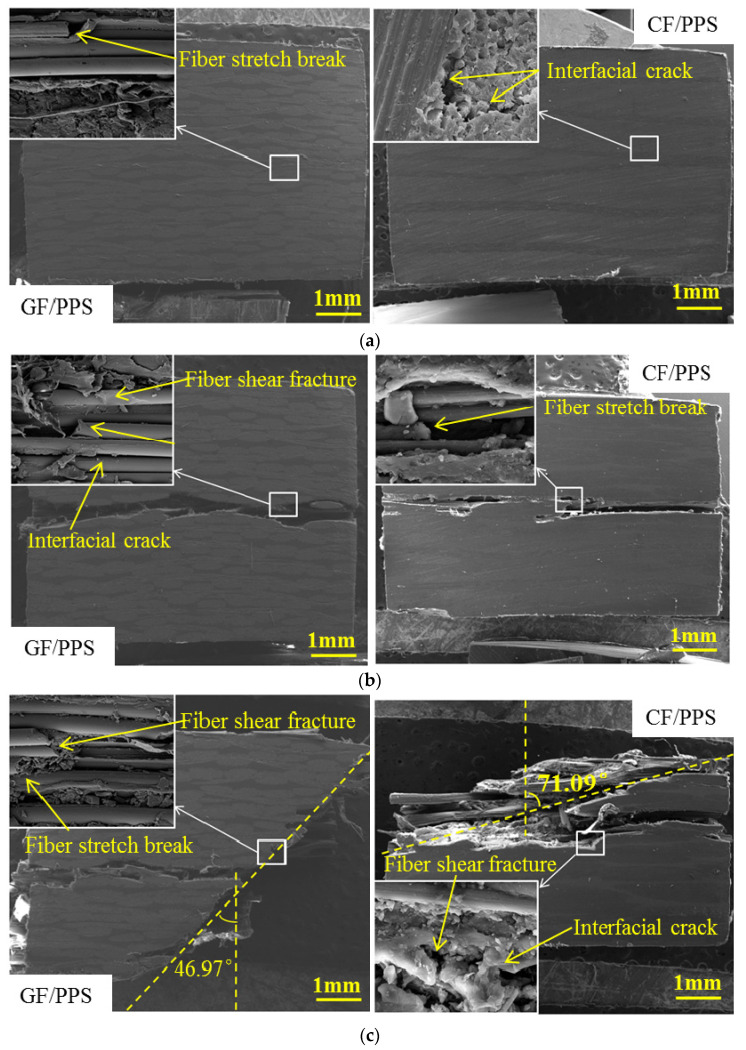

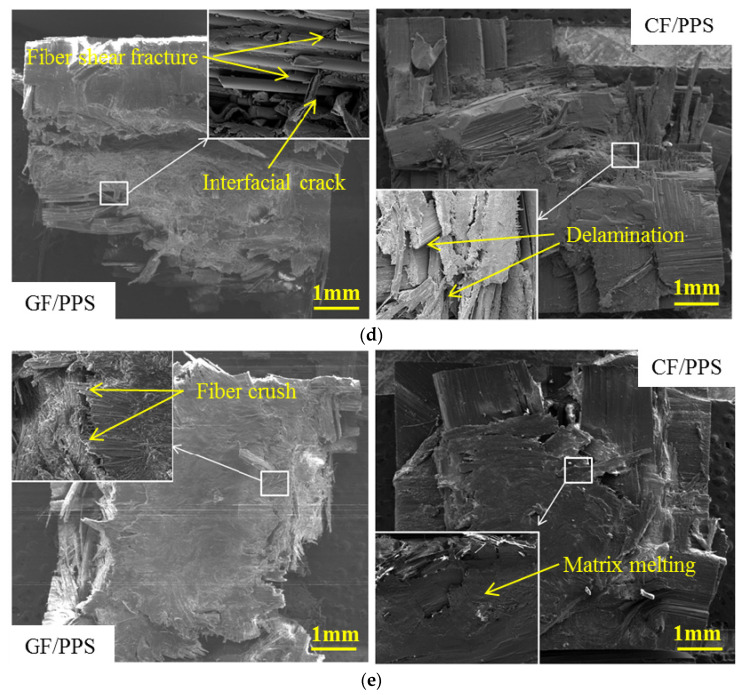
SEM micrographs of GF/PPS and CF/PPS specimens under different strain rates at room temperature: (**a**) 650/s; (**b**) 1450/s; (**c**) 1950/s; (**d**) 2450/s; (**e**) 3500/s.

In order to further analyze the failure mechanisms of the thermoplastic composites, SEM morphologies of the GF/PPS and CF/PPS specimens at RT were observed. As shown in Figure 9, the failure modes were different according to the different strain rates. At lower strain rates of 573 and 717/s, there was no visible macroscopic damage on the surface of the specimens except for a small number of interfacial cracks and stretch break of the fiber bundle caused by tensile stress at the interweaving. As the strain rate increased to 1434 and 1408/s, there was a thorough crack on the surface of the specimen. Moreover, both the stretch breakage and shear failure of the warp-fiber bundle are shown in Figure 9b, which reflects the effect of tensile stress and shear stress. This is consistent with the out-of-plane dynamic compressive failure mechanisms as described in [41].

When the strain rate exceeded the threshold, the specimen mainly had shear failure, although cracks were still found on the surface of the specimen. According to Figure 9c, the inclination angles of the failure plane of the GF/PPS and CF/PPS specimens relative to the loading axis were about 35.54° and 71.09°, respectively, which were greatly dependent on the architecture of the woven composite. At higher strain rates of 2476 and 2433/s, the characteristics of the fiber-bundle shear fracture and interlaminar cracks were clarified, which indicated that at higher strain rates, shear fracture and delamination were the main failure modes. In addition, when the strain rate increased to 3500/s, some PPS matrices of CF/PPS specimens melted during fracture. Thus, the heat generated during high-strain-rate compressive deformation may lead to failure [42].

#### 3.2.2. Effect of Temperature on Failure Mechanisms

**Figure 10:**
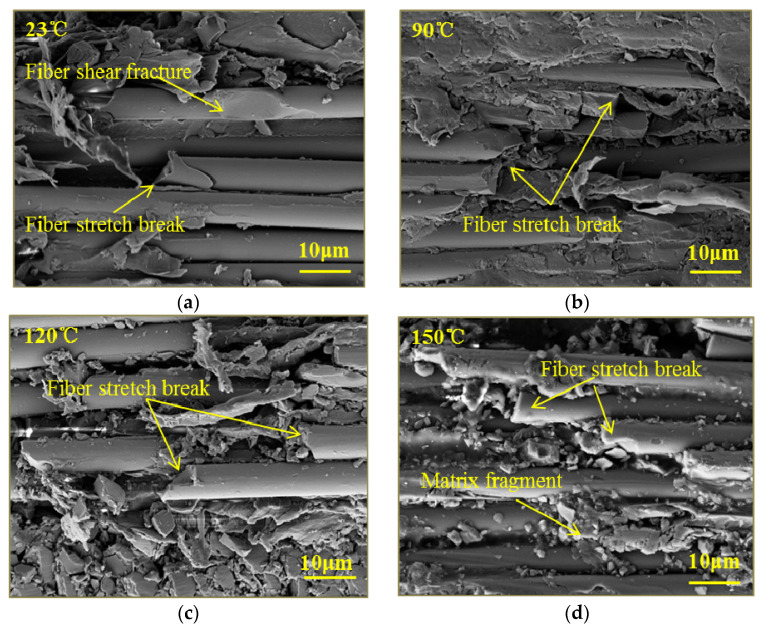
SEM micrographs of GF/PPS specimen under strain rate of 1450/s at different temperatures: (**a**) 23 °C; (**b**) 90 °C; (**c**) 120 °C; (**d**) 150 °C.

**Figure 11:**
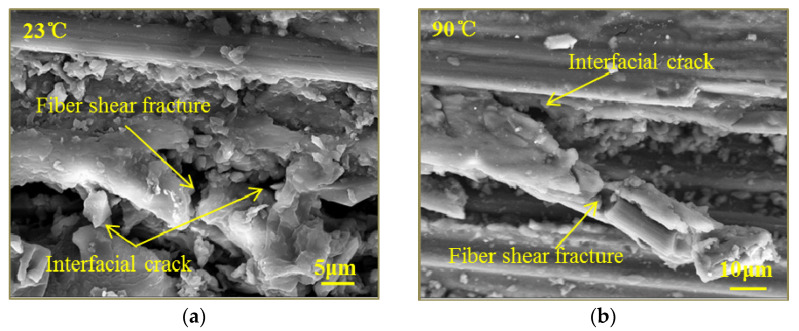

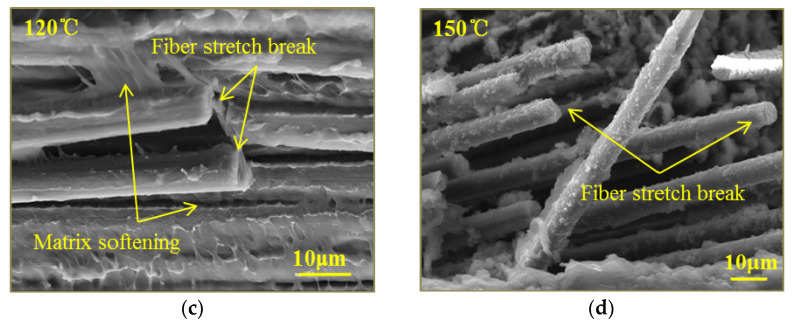
SEM micrographs of CF/PPS specimen under the strain rate of 1450/s at different temperatures: (**a**) 23 °C; (**b**) 90 °C; (**c**) 120 °C; (**d**) 150 °C.

In order to study the effect of temperature on the out-of-plane compressive failure mechanisms of woven thermoplastic composites, SEM micrographs of GF/PPS and CF/PPS specimens under a strain rate of 1450/s at different temperatures are shown in Figure 10 and Figure 11. Figure 10a and Figure 11a show that the fiber-bundle shear fracture, stretch break, and an interfacial crack were observed in the fracture of the specimens at room temperature. As temperature increased, the specimens had excellent fiber/matrix interface bonding, as shown in Figure 11c. The phenomenon mainly occurred because the plastic deformation of the matrix absorbed the energy of the stress wave and inhibited the propagation of interface cracks. Furthermore, the softening of the matrix led to a decrease in load transfer capacity. The fiber-bundle shear failure was gradually weakened, and the fiber-bundle tensile failure was the main failure mode, as shown in Figure 10d and Figure 11d. Consequently, the out-of-plane dynamic compressive strength of GF/PPS and CF/PPS composites at elevated temperatures were mainly determined by the tensile strength of the warp-fiber bundles, which were not sensitive to temperature.

## 4. Conclusions

The out-of-plane dynamic mechanical properties and damage mechanisms of thermoplastic composites at different temperatures (23–150 °C) were investigated through high-strain-rate (550–3500/s) compressive experiments. With regard to the GF/PPS and CF/PPS composites, below the threshold strain rate, strength, modulus, and failure strain increased with the increase in strain rate, and the strain-rate-sensitivity (SRS) indicators of the two thermoplastic composites were almost the same. Beyond the threshold strain rate, the mechanical properties of the CF/PPS composite continued to linearly increase, but the strength of GF/PPS composite slowly increased. The softening of the matrix at elevated temperatures lead to a decrease in the stiffness of GF/PPS and CF/PPS composites and an increase in plastic deformation, but had the little effect on out-of-plane compressive strength. CF/PPS composites exhibited greater temperature dependence.

Under out-of-plane dynamic compressive load, the specimen was subjected to the combined effect of tensile stress caused by the Poisson effect and shear stress at the yarn interlacing. As strain rate increased, the shear fracture of the fiber bundle caused by the shear stress gradually became the main failure mode. As the temperature increased, the fiber-bundle stretch break caused by the tensile stress became the main failure mode.

## Figures and Tables

**Table 1:** Dynamic compression properties for GF/PPS and GF/PPS composites at RT.

GF/PPS Composite				CF/PPS Composite			
Strain Rate (s-1)	Strength (MPa)	Modulus (GPa)	Failure Strain (%)	Strain Rate (s-1)	Strength (MPa)	Modulus (GPa)	Failure Strain (%)
573	414.87	12.94	3.94	717	354.96	11.17	3.52
1434	598.38	13.83	7.04	1408	441.05	11.67	4.48
1980	653.77	14.49	8.19	1934	494.75	11.99	5.34
2476	664.16	14.77	8.11	2433	539.33	12.34	6.21
3520	685.57	15.33	8.03	3414	625.27	12.85	7.94

**Table 2:** Parameters of strain rate dependence on dynamic compression properties for GF/PPS and GF/PPS composites.

Specimen	Strength (σ)		Modulus (E)		$\mathrm{Failure}\;\mathrm{Strain}\;(\varepsilon_{u})$	
	$K_{\sigma }$	m	$K_{E}$	m	$K_{\varepsilon }$	m
GF/PPS	40.18	0.369	8.31	0.087	0.11	0.567
CF/PPS	30.37	0.367	6.38	0.088	0.075	0.569

**Table 3:** Dynamic compression properties for GF/PPS and GF/PPS composites at different temperatures.

Temperature (°C)	GF/PPS Composite			CF/PPS Composite		
	Strength (MPa)	Modulus (GPa)	Failure Strain (%)	Strength (MPa)	Modulus (GPa)	Failure Strain (%)
23	598.38	13.82	7.04	441.05	11.67	4.48
90	596.51	13.28	7.27	425.11	10.18	5.17
120	603.43	12.17	7.67	410.18	7.16	5.81
150	601.09	11.41	8.65	355.72	6.41	6.73

**Table 4:** Mean absolute percentage errors (*MAPE*) of predicted mechanical properties of thermoplastic composites.

Materials	Strength (%)	Modulus (%)	Failure Strain (%)
GF/PPS	1.49	0.78	1.17
CF/PPS	1.95	2.46	1.76

## Data Availability

The data presented in this study are available on request from the corresponding author.
